# Fibrillatory Wave Amplitude Evolution during Persistent Atrial Fibrillation Ablation: Implications for Atrial Substrate and Fibrillation Complexity Assessment

**DOI:** 10.3390/jcm11154519

**Published:** 2022-08-03

**Authors:** Fabien Squara, Didier Scarlatti, Sok-Sithikun Bun, Pamela Moceri, Emile Ferrari, Olivier Meste, Vicente Zarzoso

**Affiliations:** 1 Cardiology Department, Université Côte d’Azur, Pasteur Hospital, 30 Avenue de la Voie Romaine, 06000 Nice, France; scarlatti.d@chu-nice.fr (D.S.); bun.s@chu-nice.fr (S.-S.B.); moceri.p@chu-nice.fr (P.M.); ferrari.e@chu-nice.fr (E.F.); 2 I3S Laboratory, Université Côte d’Azur, CNRS, 06900 Sophia Antipolis, France; olivier.meste@univ-cotedazur.fr (O.M.); vicente.zarzoso@univ-cotedazur.fr (V.Z.)

**Keywords:** atrial fibrillation, ECG, ablation

## Abstract

**Background**. Fibrillatory Wave Amplitude (FWA) has been described as a non-invasive marker of atrial fibrillation (AF) complexity, and it predicts catheter ablation outcome. However, the actual determinants of FWA remain incompletely understood. **Objective**. To assess the respective implications of anatomical atrial substrate and AF spectral characteristics for FWA. **Methods**. Persistent AF patients undergoing radiofrequency catheter ablation were included. FWA was measured on 1-min ECG by TQ concatenation in Lead I, V1, V2, and V5 at baseline and immediately before AF termination. FWA evolution during ablation was compared to that of AF dominant frequency (DF) measured by Independent Component Analysis on 12-lead ECG. FWA was compared to the extent of endocardial low-voltage areas (LVA I < 10%; II 10–20%; III 20–30%; IV > 30%), to the surface of healthy left atrial tissue, and to P-wave amplitude in sinus rhythm. The predictive value of FWA for AF recurrence during follow-up was assessed. **Results**. We included 29 patients. FWA remained stable along ablation procedure with comparable values at baseline and before AF termination (Lead I *p* = 0.54; V1 *p* = 0.858; V2 *p* = 0.215; V5 *p* = 0.14), whereas DF significantly decreased (5.67 ± 0.68 vs. 4.95 ± 0.58 Hz, *p* < 0.001). FWA was higher in LVA-I than in LVA-II, -III, and -IV in Lead I and V5 (*p* = 0.02 and *p* = 0.01). FWA in V5 was strongly correlated with the surface of healthy left atrial tissue (R = 0.786; *p* < 0.001). FWA showed moderate to strong correlation to P-wave amplitude in all leads. Finally, FWA did not predict AF recurrence after a follow-up of 23.3 ± 9.8 months. **Conclusions**. These findings suggest that FWA is unrelated to AF complexity but is mainly determined by the amount of viable atrial myocytes. Therefore, FWA should only be referred as a marker of atrial tissue pathology.

## 1. Introduction

Atrial fibrillation (AF) is the most frequently encountered cardiac arrhythmia and is responsible for significant morbidity and mortality [1]. Based on the duration of the arrhythmia episodes, different AF types have been described: paroxysmal (<7 days), persistent (>7 days), and long-standing persistent AF (>1 year). Empirical pulmonary vein isolation remains the cornerstone of the ablation strategy [1]; however, many additional substrate-based ablation strategies have been developed for improving ablation results in persistent and long-standing persistent AF, but the most appropriate target remains debated.

Despite important improvements since its initial description, catheter ablation of AF remains complex and time-consuming, and appropriate selection of candidates for ablation is critical. The time and frequency domains analyses of the fibrillatory wave on the surface ECG has gained increasing interest over the past decades for predicting catheter ablation outcomes, which can help in determining the most appropriate treatment for patients with persistent and long-standing persistent AF [2].

Fibrillatory wave amplitude (FWA) was one of the first ECG-derived parameters to be evaluated for assessing AF complexity, and previous studies found that patients with lower FWA presented more frequent ablation failures, because of a presumably higher degree of AF complexity [3,4,5,6]. FWA on the surface ECG is dependent on the magnitude of the underlying atrial voltage, which is assumed to be determined by two factors: (1) the amount of remaining viable atrial muscle, and (2) the number of waves and breakthroughs during AF [7], which can modulate voltage by cancellation of colliding activations. However, the actual participation of those two factors remains unknown and, accordingly, it is unclear if FWA is truly able to depict AF complexity or is only a marker of anatomical atrial disease.

In this study, we sought to evaluate the links of FWA with anatomical atrial substrate and AF complexity by assessing the evolution of FWA during radiofrequency catheter ablation of persistent and long-standing persistent AF. We also compare FWA to AF dominant frequency, to the amount of left atrial (LA) low voltage areas and of healthy LA tissue, and to P-wave voltage after sinus rhythm recovery.

## 2. Methods

### 2.1. Study Population

Consecutive patients undergoing first time radiofrequency ablation for persistent or long-standing persistent symptomatic drug-refractory AF at Pasteur University Hospital, Nice, France, were included. The study was approved by the institutional review board, all patients gave written informed consent, all methods were performed in accordance with the relevant guidelines and regulations, and research was performed in accordance with the declaration of Helsinki.

### 2.2. Electroanatomical Mapping and Ablation Procedure

Procedures were performed under general anesthesia. Transesophageal echocardiography (TEE) was performed before venous puncture for ruling out left atrial appendage (LAA) thrombus. A decapolar catheter was positioned in the coronary sinus (CS), through a transfemoral venous access under vascular echography guidance. A single or double transseptal puncture was performed under TEE guidance. A bolus of unfractionated heparin was administered immediately after the transseptal puncture and infusion was titrated to maintain activated clotting time >300 s for the duration of the procedure. Procedures were performed without interruption of vitamin K antagonists with International Normalized Ratio < 3, and new oral anticoagulants were interrupted the day before the procedure.

High-density electroanatomical mapping (>1000 points) of the entire LA was performed with the Carto system V6 or V7 (Biosense Webster Inc., Irvine, CA, USA), using the Pentaray catheter (Biosense Webster Inc.) inserted transseptally via a non-steerable sheath (Fast-Cath SL0; St. Jude Medical, Minnetonka, MN, USA). The Thermocool SmartTouch SF ablation catheter (Biosense Webster Inc.) was inserted through the transseptal puncture in another SL0 sheath. Detailed mapping of electrical activation of the LA (and, in selected cases, of the right atrium) was performed, with visual annotation of zones of spatiotemporal electrogram dispersion (STD) [8] and of continuous complex fractionated atrial electrograms (CFAEs) [9]. At baseline, AF cycle length was measured in the LAA by averaging 10 cycles. Voltage mapping of the LA was performed in AF before ablation. A bipolar cut-off of 0.25 mV was used for defining pathological tissue, because scar thus delineated demonstrated to correlate well with scar identified by a bipolar voltage <0.5 mV in sinus rhythm [10]. The extent of LA endocardial low-voltage areas (LVA) was then assessed, and four categories were distinguished: LVA-I (<10% of low-voltage areas compared to LA surface), LVA-II (10–20%), LVA-III (20–30%), and LVA-IV (>30%). These values were chosen in line with the UTAH stages assessed by MRI [11].

LA volume (mL) and LA surface (cm^2^) were assessed using high-density electroanatomical mapping with the Carto system, after excluding the inside portion of PVs and the mitral valve. The surface of healthy atrial tissue was determined by subtracting the surface of LVA to the total LA surface.

Point-by-point ablations were carried out in a stepwise manner, with endpoints of circumferential pulmonary vein (PV) disconnection, ablation at STD and CFAE sites, and block across the lines (if performed). Roof lines, anterior mitral lines, or lateral mitral lines were performed in cases of left atrial substrate in the area (presence of STD or CFAEs). There was no recommendation regarding the order of the treated LA areas, and ablation could be initiated either by PV isolation or by left atrial substrate ablation. Each area was strictly ablated in a sequential manner, for allowing assessment of the effect of each area ablation on AF complexity parameters. LA areas were defined as: left PVs ostia, right PVs ostia, roof, anterior, septal, floor, lateral isthmus, and LAA base.

High-power short duration irrigated radiofrequency was delivered using a power of 50 W and with an ablation index target of 350 arbitrary units (AU) on the posterior wall of the LA, and 420 AU elsewhere. If CS ablation was performed, radiofrequency power was limited to 25–30 W.

AF termination during ablation was defined as sinus rhythm resumption or its change to a stable atrial tachyarrhythmia. Nevertheless, this was not a procedural endpoint, and operators ended the procedures after PV isolation, complete ablation of the annotated STD and CFAE sites, and, when appropriate, block across the lines. Associated atrial tachyarrhythmia ablation was performed, and the critical isthmus or focal origin was specifically targeted up to sinus rhythm resumption and reconfirmation of PV isolation before catheter withdrawal. In cases without AF or atrial tachyarrhythmia termination at the end of the procedure, an electrical cardioversion was performed (200 J biphasic, repeated up to three times).

### 2.3. Follow-Up

A blanking period of 1 month was chosen in this study, because early arrhythmia recurrences at 1–3 months have proven to be predictive of late arrhythmia recurrences [12,13,14]. After this 1-month blanking period, patients were followed for clinical and asymptomatic recurrences for at least 12 months. Follow-up was performed in a real-life setting, by regular visits to the treating cardiologist, with repeated ECG and 24-h Holter monitoring in all cases (every 3 months during the first year after ablation; every 6 months afterwards). Supplementary documentation by ECG or Holter was sought in the case of recurring symptoms suggestive of arrhythmia. Recurrence of sustained (>30 s) AF or flutter was recorded. In the absence of arrhythmia recurrence, antiarrhythmic drugs were stopped 1 month after ablation.

### 2.4. Signal Acquisition

ECG signals were acquired on a digital electrophysiological recording system (Labsystem Pro EP, Boston Scientific, Marlborough, MA, USA), including 0.05 to 40 Hz band pass and 50 Hz notch filters. For every patient, a 1-min standard 12-lead ECG in AF was recorded at a sampling rate of 1000 Hz at the beginning of the procedure, after each LA area ablation, and immediately before AF termination (either per-radiofrequency termination, or termination by electrical cardioversion). Additionally, a 1-min standard 12-lead ECG was recorded in sinus rhythm at the end of the procedure.

### 2.5. Signal Processing

#### 2.5.1. F-Wave and P-Wave Amplitude Computation

A previous study designated Leads I, V1, V2, and V5 as the most discriminant to determine mid- and long-term catheter ablation outcome based on FWA [6], and therefore only those leads were considered in subsequent analyses. FWA was measured in Leads I, V1, V2, and V5 at baseline and immediately before AF termination. In lead V1, FWA was also measured after each LA area ablation.

**Figure 1:**
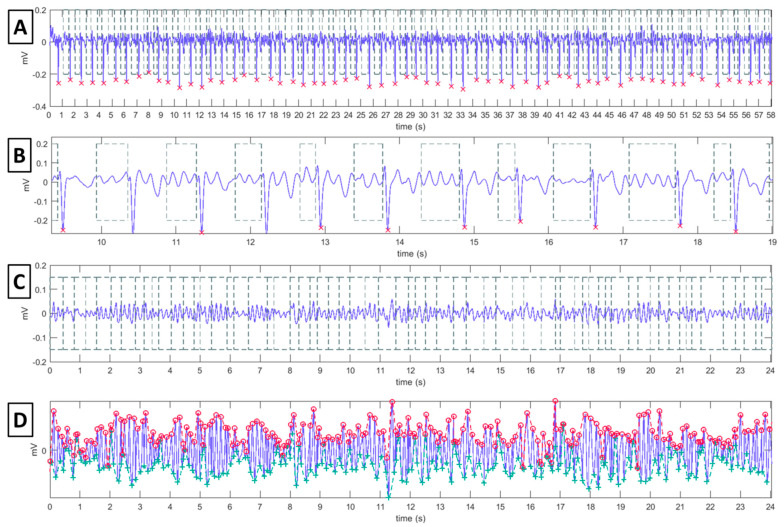
F-wave amplitude measurement using TQ-segment concatenation technique. (Panel **A**): Electrocardiogram in Lead V1 with detection of the R-waves peaks (red crosses) as well as the Q-wave onset and T-wave offset locations, and segmentation of the TQ intervals (dashed boxes). To ease visualization, 10 s of this recording are shown in (panel **B**). (Panel **C**): The TQ segments (dashed boxes) are mean centered and concatenated. (Panel **D**): Local maxima (red circles) and minima (green crosses) are detected in the concatenated TQ segments, and then interpolated to yield an estimate of the upper and lower envelopes (red and green dashed line, respectively) of the atrial activity signal; at each time instant, the difference between the two envelopes provides an instantaneous estimate of the f-wave amplitude in the ECG lead considered.

The outcomes of the signal processing algorithm used to compute the FWA in each lead is illustrated in Figure 1. We have previously described the mathematical details of this amplitude measurement algorithm [6,15]. First, ECG fiducial points were detected to properly segment TQ intervals, where atrial activity can be measured free from QRST complexes of ventricular interference. R-wave time instants were located on lead V1 by applying the Pan–Tompkins algorithm [16], and Q-wave onsets were obtained by subtracting 40 to 80 ms, depending on the QRS width. From the lead where the most prominent T-waves could be visually identified, T-wave offsets were estimated by an adapted Woody’s method [17]; the segmented intervals were then mean centered and concatenated. In the concatenated TQ segments, the local maxima were detected, and an upper envelope was estimated by interpolation; the lower envelope was estimated in an analogous manner from the local minima. Finally, the average difference between both envelopes along time was computed as an estimate of the f-wave mean amplitude in the lead examined. Amplitude computation was performed using MATLAB, version 2020b (MathWorks, Natick, MA, USA).

Once in sinus rhythm at the end of the procedure, P-wave amplitude was measured. Because P-wave is stable over time, its amplitude was assessed by peak-to-peak measurement.

#### 2.5.2. AF Dominant Frequency Assessment

AF dominant frequency (DF) is related to the refractory period atrial myocardium cells, and thus to the degree of complexity of the disease and the probability of spontaneous cardioversion [18]. In this work, we assessed DF at every step of the ablation procedure by means of principal component analysis (PCA) and robust independent component analysis (RobustICA) on a 12-lead ECG. This approach to atrial activity extraction relies on the observation that atrial activity and ventricular activity can be considered a statistically independent phenomena [19]. Techniques for the separation of independent signals such as PCA and RobustICA can then be applied on the 12-lead ECG to search for the atrial activity source, thus allowing the reconstruction of atrial activity in all leads free from ventricular activity and other interference. Details on the technique have been given previously [20,21]. We favored DF measurement over sequential LAA cycle assessment because regional radiofrequency ablation in the vicinity of LAA can affect the LAA cycle, and as such, it might not reliably reflect AF complexity evolution during the ablation procedure.

**Figure 2:**
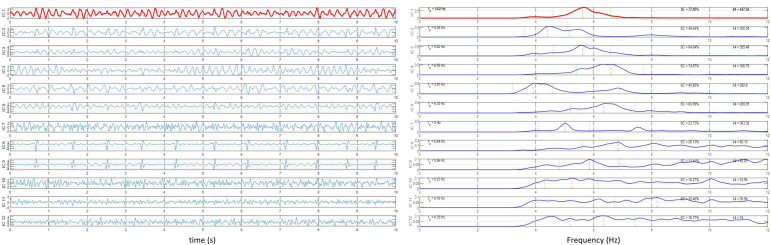
Signal processing for dominant frequency measurement using RobustICA. (**Left panel**): Separation of the independent signals acquired from the 12-lead ECG; to ease visualization, only 10 s of the recordings are shown. (**Right panel**): Frequency spectra of the signals shown in the left panel. The atrial activity source is identified as the extracted component with dominant frequency in the interval [3–9] Hz with the highest spectral concentration value. In both panels, the identified atrial activity source is displayed in red.

The atrial activity source is automatically selected as the extracted component with a dominant peak in the interval [3–9] Hz, the typical AF frequency band. The percentage of signal power around the dominant peak, or spectral concentration (SC), has been shown to correlate with atrial activity extraction quality [22] and is hence used as a measure of performance. PCA and RobustICA were implemented using MATLAB, version 2020b (MathWorks). Figure 2 illustrates the signal processing for DF measurement using RobustICA.

### 2.6. Statistical Analysis

Statistical analyses were performed using SPSS Statistics v20 software (IBM, Armonk, NY, USA). Distribution normality of continuous variables was checked with the Kolmogorov—Smirnov test. Categorical variables were presented as percentages and continuous variables as mean and standard deviation in the case of distribution normality, or median and confidence interval otherwise. Under data normality, groups were compared by a parametric unpaired or paired *t*-test as appropriate, whereas a non-parametric Mann—Whitney *U*-test was used when the variables did not show a normal distribution. When more than two groups were present, ANOVA analysis was performed. Proportion analysis was based on the chi-square test. Correlation between continuous variables was performed using the Pearson’s R coefficient. Statistical significance was defined by a *p* value < 0.05.

## 3. Results

We included 29 patients (age 67.9 ± 9.2 years; 27.6% females). Clinical and procedural characteristics are given in Table 1. Patients had failed antiarrhythmic therapy with amiodarone (18/29; 62%) or Flecainide (11/29; 38%). Mean AF duration was 21.7 ± 20.4 months, and 15/29 patients (51.7%) had a long-standing persistent AF (>12 months). AF cycle length measured in the LAA at baseline was 169.7 ± 37.1 ms. We performed a high resolution electroanatomical mapping of the LA substrate with 3171 ± 1864 points acquired with the Carto system. We documented 5.5 ± 1.5 atrial zones of STD or continuous CFAEs per patient. The total surface of LA zones with STD or CFAEs was 21.3 ± 8.7 cm^2^ per patient, representing 8.9 ± 3.6% of the total LA surface.

### 3.1. F-Wave Amplitude Evolution and Correlation with AF Complexity Parameters

Baseline FWA was neither correlated with LAA cycle length (Lead I *p* = 0.49; Lead V1 *p* = 0.59; Lead V2 *p* = 0.71; and Lead V5 *p* = 0.75) nor with DF using RobustICA or PCA (Lead I *p* = 0.8 and *p* = 0.95; Lead V1 *p* = 0.69 and *p* = 0.73; Lead V2 *p* = 0.52 and *p* = 0.5; Lead V5 *p* = 0.67 and *p* = 0.6). However, baseline DF was strongly inversely correlated with LAA cycle length (R = −0.78, *p* < 0.001 using RobustICA; R = −0.79, *p* < 0.001 using PCA).

FWA remained stable along the ablation procedure in all studied leads; baseline FWA was similar to FWA before AF termination in Lead I (13.6 ± 5.7 vs. 12.7 ± 4.9 µV; *p* = 0.535), Lead V1 (47.4 ± 19.3 vs. 46.4 ± 20.8 µV; *p* = 0.858), Lead V2 (39.5 ± 13.0 vs. 35.2 vs. 13.4 µV; *p* = 0.215), and Lead V5 (23.9 ± 8.4 vs. 20.6 ± 8.7 µV; *p* = 0.14). These results remained unchanged when only the patients whose AF terminated during radiofrequency application were analyzed (19/29 patients; 65.5%).

In contrast, DF significantly decreased during ablation using RobustICA (5.67 ± 0.68 vs. 4.95 ± 0.58 Hz; *p* < 0.001) and PCA (5.70 ± 0.68 vs. 5.0 ± 0.55 Hz; *p* < 0.001). Spectral concentration value was higher using RobustICA than using PCA (69.4 ± 9.7 vs. 58.6 ± 11.5%; *p* < 0.001), evidencing the superior atrial activity source separation using RobustICA and suggesting the better performance of this technique for DF assessment.

**Figure 3:**
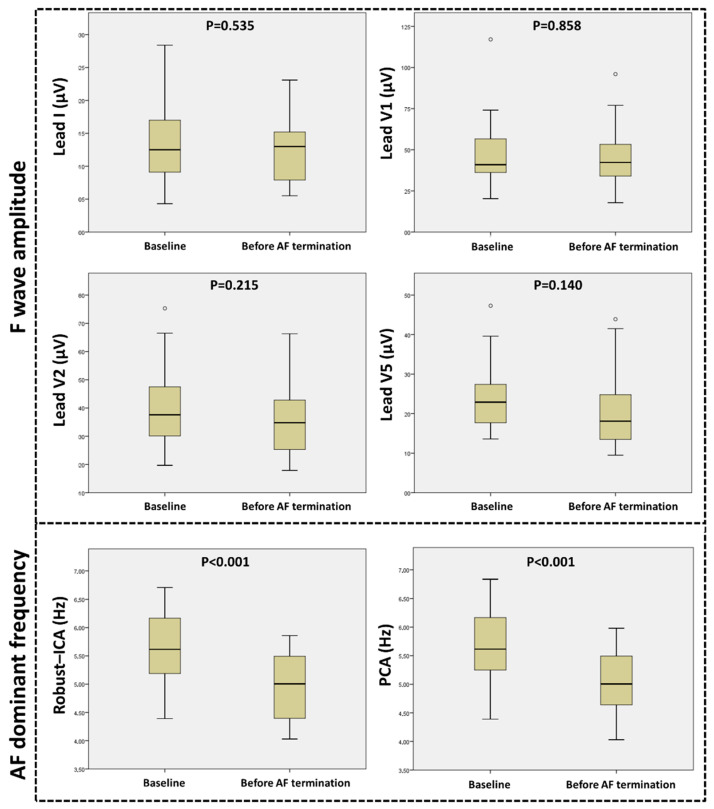
Box plot diagrams showing the evolution during the ablation procedure of the f-wave amplitude in the four studied leads (**top panel**), and of the AF dominant frequency using RobustICA and PCA (**bottom panel**). *p* values were calculated using the paired *t*-test.

Figure 3 illustrates the evolution of FWA and DF from baseline to end-procedure.

### 3.2. Relationship between ECG Parameters and the Extent of Anatomical LA Substrate

There was a strong correlation between the surface of healthy LA tissue and baseline FWA in lead V5 (R = 0.786; *p* < 0.001). There was also a significant correlation with baseline FWA in Lead I and Lead V2, but with a weaker correlation coefficient (R = 0.394, *p* = 0.035; and R = 0.486, *p* = 0.007). No correlation was evidenced with FWA in lead V1 (*p* = 0.178).

**Figure 4:**
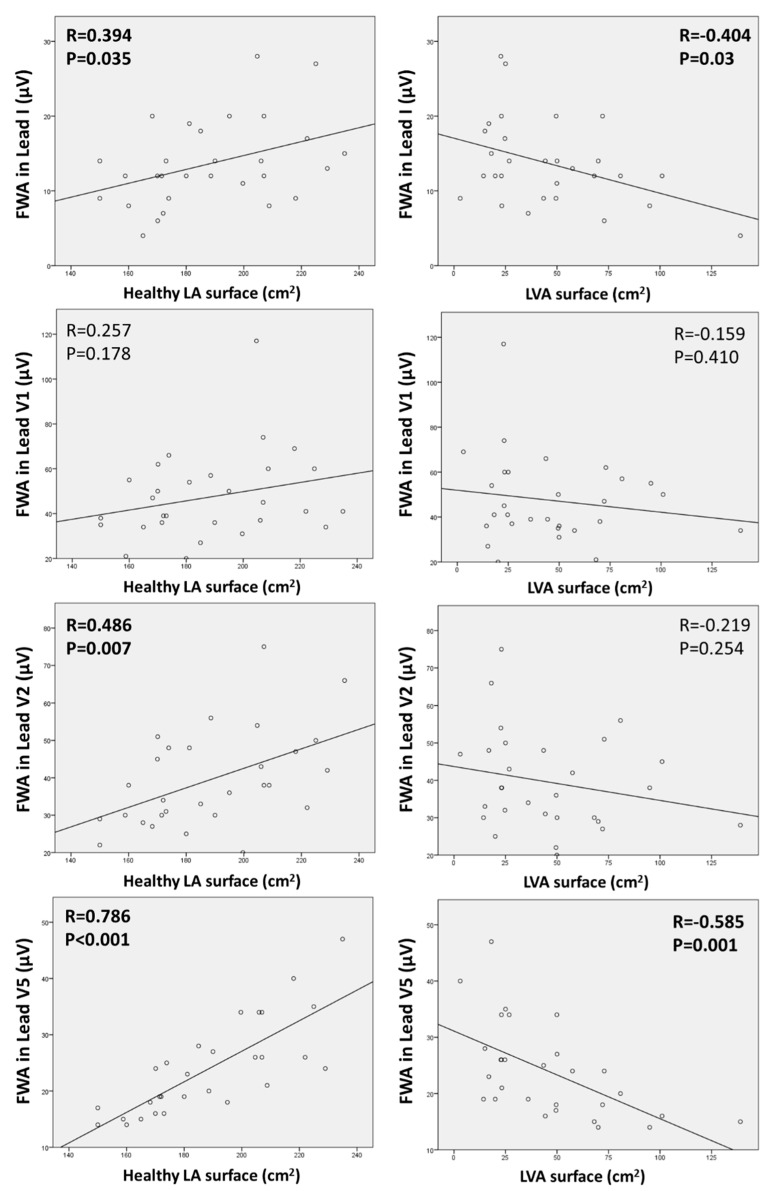
Scatterplots showing the correlations between baseline f-wave amplitude in the studied leads and healthy left atrial surface (**left**) or low-voltage area surface (**right**). The best correlation is seen with Lead V5, due to its localization on the left precordium and thus better displaying left atrial electrical activity. Correlations were performed using the Pearson’s R coefficient.

The mean surface of LVA was 46 ± 31.4 cm^2^, representing 18.9 ± 11.2% of the total LA surface. The surface of LVA was moderately inversely correlated with baseline FWA in lead I (R = −0.404; *p* = 0.03) and in Lead V5 (R = −0.585; *p* = 0.001). See Figure 4 for details.

**Figure 5:**
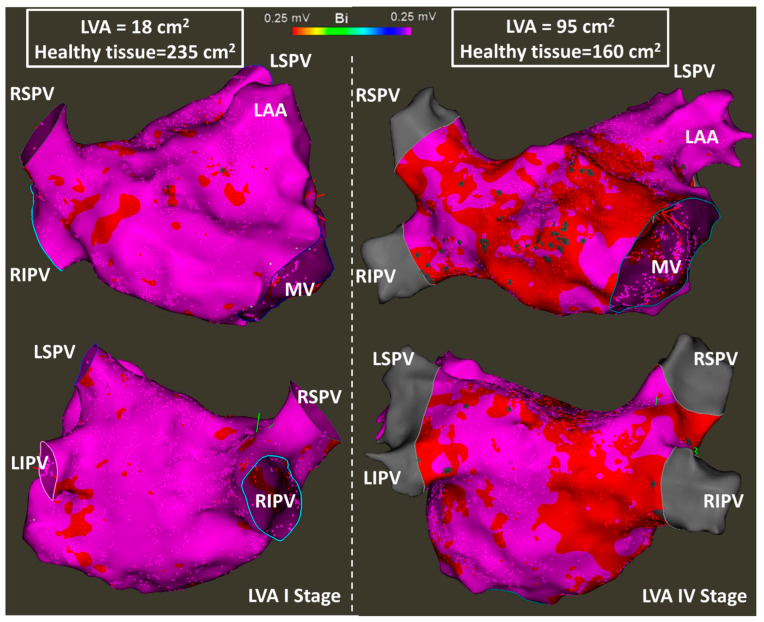
Electroanatomical mapping with delineation of the LA low-voltage areas using bipolar voltage <0.25 mV. The left panel shows an LA with a low scar burden (LVA I), whereas the right panels shows an LA with a high scar burden (LVA IV).

Amongst the 29 patients, 12 (41.4%) were LVA-I (<10% of low-voltage areas compared to LA surface), 5 (17.2%) were LVA-II (10–20% of low-voltage areas), 4 (13.8%) were LVA-III (20–30% of low-voltage areas), and the remaining 8 (27.6%) were LVA-IV (>30% of low-voltage areas). Figure 5 shows comparative examples of LVA-I and LVA-IV patients.

FWA in Lead V5 was significantly correlated with LVA stages (*p* = 0.007), and there was a trend towards a correlation for Lead I (*p* = 0.1). For Leads V1 and V2, no link was evidenced between FWA and LVA stages (*p* = 0.6 and *p* = 0.53, respectively). FWA was significantly higher in patients with LVA-I stage compared to LVA stage ≥II in Lead V5 (28.7 ± 8.7 vs. 20.6 ± 6.4 µV; *p* = 0.008) and in Lead I (16.4 ± 6.4 vs. 11.7 ± 4.4 µV; *p* = 0.026), but not in Leads V1 and V2 (*p* = 0.15 and *p* = 0.072, respectively). However, LVA stage was not related to DF (*p* = 0.81 for RobustICA; *p* = 0.77 for PCA), AF duration (*p* = 0.81), or LA volume (*p* = 0.35).

After sinus rhythm resumption, P-wave amplitude was moderately correlated with the surface of healthy LA tissue in Lead V1 (R = 0.473; *p* = 0.01), Lead V2 (R = 0.432; *p* = 0.019), and Lead V5 (R = 0.482; *p* = 0.008). No significant correlation was seen with Lead I (*p* = 0.16).

### 3.3. Relation between F-Wave Amplitude and P-Wave Amplitude

**Figure 6:**
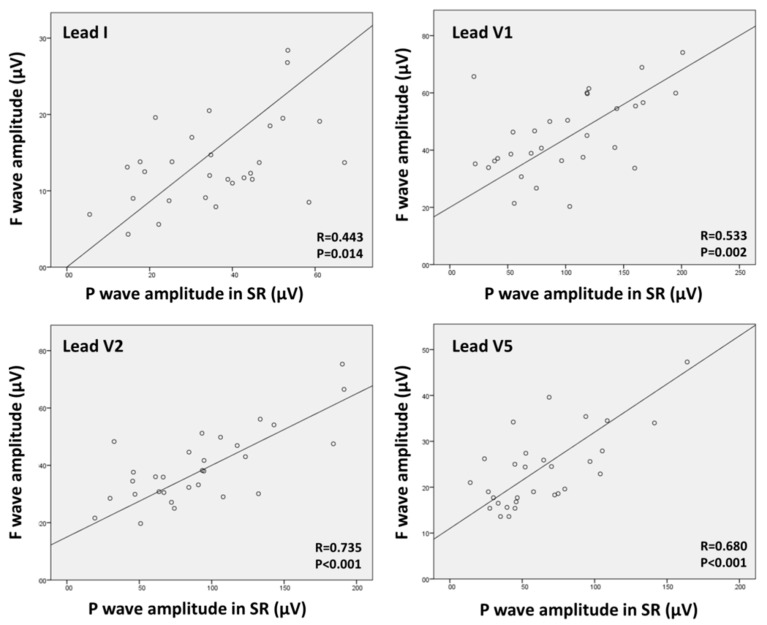
Scatterplots showing the correlation between f-wave amplitude in atrial fibrillation and P-wave amplitude in sinus rhythm. Correlations were performed using the Pearson’s R coefficient.

P-wave amplitude after sinus rhythm resumption was significantly correlated with baseline FWA in all studied leads, with a moderate to strong correlation coefficient (Lead I R = 0.443, *p* = 0.014; Lead V1 R = 0.533, *p* = 0.002; Lead V2 R = 0.735, *p* < 0.001; Lead V5 R = 0.680; *p* < 0.001). Figure 6 shows the scatterplots of P-wave amplitude and FWA.

### 3.4. AF Termination and Follow-Up

AF termination during radiofrequency was obtained in 19/29 patients (65.5%); the remaining 10 patients underwent electrical cardioversion at the end of the procedure. Baseline DF was lower in patients for whom AF termination was reached per ablation as compared to those who underwent electrical cardioversion at the end of the procedure (5.49 ± 0.66 vs. 6.02 ± 0.59 Hz; *p* = 0.042), as well as AF duration (15.7 ± 12.5 vs. 34.3 ± 27.9 months; *p* = 0.021) and LA volume (158 ± 31 vs. 192 ± 38 mL; *p* = 0.014). However, baseline FWA was similar in both groups: 14.2 ± 6.5 vs. 12.4 ± 3.7 µV in Lead I (*p* = 0.44), 48.9 ± 22.5 vs. 44.4 ± 11.4 µV in Lead V1 (*p* = 0.56), 39.6 ± 14.1 vs. 39.4 ± 11.5 µV in Lead V2 (*p* = 0.97), and 22.3 ± 6.9 vs. 27.1 ± 10.5 µV in Lead V5 (*p* = 0.18). The surface of healthy LA tissue and the surface of LVA were not linked with AF termination (*p* = 0.109 and *p* = 0.324).

After a follow-up of 23.3 ± 9.8 months, 2/29 patients (6.9%) had AF recurrence and 11/29 patients (37.9%) demonstrated left atrial flutter; 16/29 (55.2%) remained free from any arrhythmia after a single ablation procedure. Patients with recurring AF had a longer AF duration before ablation (72 (48–96) vs. 12 (6–27.3) months; *p* = 0.0221), a larger LA (238 (226–251) vs. 165 (141–177) ml; *p* = 0.0252) and a higher DF (6.53 (6.47–6.59) vs. 5.62 (5.24–6.09) Hz; *p* = 0.0371). However, FWA was similar in recurring AF or non-recurring AF patients in Lead I (11.4 (9.1–13.7) vs. 12.8 (11.2–16.0) µV; *p* = 0.5467), Lead V1 (51.4 (37.1–65.7) vs. 40.8 (36.2–52.6) µV; *p* = 0.3438), Lead V2 (45.7 (43.0–48.3) vs. 35.2 (30.3–43.3) µV; *p* = 0.7963) and Lead V5 (29.8 (25.0–34.5) vs. 22.0 (18.0–26.1) µV; *p* = 0.1212). The surface of healthy LA tissue and the surface of LVA were not correlated with AF recurrence (*p* = 0.572 and *p* = 0.728).

## 4. Discussion

### 4.1. Main Results

In this study, we assessed FWA during persistent AF ablation and compared it to its assumed determinants: the degree of AF complexity and the amount of viable LA tissue.

Our results suggest that FWA is unrelated to AF complexity. First, at baseline, FWA did not demonstrate any correlation with DF or LAA cycle length, which are established parameters of AF complexity. Second, FWA was stable in all leads along the ablation procedure, with similar values at baseline, where AF is the most complex, and immediately before AF termination or cardioversion, where AF is assumed to be the least complex. In contrast, DF demonstrated a significant decrease during the ablation procedure. Finally, FWA was neither correlated with AF termination during the ablation procedure, nor with AF recurrence during follow-up, whereas DF, LA volume, and AF duration were correlated with both.

On the other hand, FWA was significantly correlated with the amount of LA low-voltage areas and with the surface of healthy LA tissue, with the best results for lead V5, which better displays LA electrical activity due to its localization on the left precordium. Additionally, FWA was correlated in all studied leads with P wave amplitude in sinus rhythm, which is fundamentally uncomplex and is directly related to atrial tissue quality.

All these findings point to the fact that FWA is a marker of atrial tissue pathology, but not a direct parameter of AF complexity.

### 4.2. Previous Studies and Purpose of FWA Assessment

Several previous studies have assessed the value of FWA for predicting AF recurrence after catheter ablation, using manual [3,5] or automated [4,6,23,24] approaches, but important inconsistencies in the results raise concern. Using single-lead FWA analyses, Nault et al. [5] and Cheng et al. [3] both found FWA in lead V1 to be lower in recurring AF patients, whereas Henricksson et al. [23] found no difference, and in a study by Zarzoso et al. [6], FWA was similar in all leads except for Lead I, which demonstrated higher FWA in recurring patients. The latter study also performed multiple-lead FWA analysis with encouraging results, and pointed to leads I, V1, V2, and V5 as the most discriminant to determine catheter ablation outcomes. In our present study, those four leads were analyzed individually, and none of them demonstrated significant differences in recurring patients, or in patients undergoing electrical cardioversion at the end of the procedure. Taken together, all these results highlight the difficulty of translating FWA measurement into routine clinical practice.

During catheter ablation of AF, a progressive organization occurs and AF complexity gradually decreases [25,26]. As such, it would be expected that FWA progressively increases during the course of the ablation procedure. However, in three previous studies that assessed the impact of each ablation step on complexity parameters, two of them did not demonstrate any changes in FWA during ablation, and one even found a significant decrease in lead V6, whereas DF constantly decreased [4,23,24]. These results are consistent with ours, and further indicate that FWA is not correlated with AF complexity, and that disorganization implying multiple activation fronts in different directions does not have a significant effect on electrical vectors producing the f-wave on the ECG. However, similarly to the P-wave amplitude [27], our study demonstrated that FWA is directly dependent on the amount of left atrial scar and of the remaining healthy atrial tissue. Thus, in ECG leads predominantly displaying LA electrical activity—such as Lead I, V5, and V6—a decrease in FWA after catheter ablation could be easily explained by the reduction of the LA viable tissue, especially after extensive ablation procedures. Our relatively conservative approach (STD or CFAEs representing 8.9 ± 3.6% of the total LA surface) might explain the stability of post-ablation FWA in our study.

As a marker of atrial tissue pathology, FWA assessment remains valuable for predicting complex ablation procedures, because AF genesis and maintenance is strongly linked with atrial fibrosis [28]. However, aside from some anecdotal cases, reliable FWA assessment requires computing skills, and no clear voltage cut-off has been determined for predicting ablation failure, therefore limiting its widespread use.

### 4.3. Limitations

Our group of patients is relatively small, with 29 patients included. With a larger number of patients, it is possible that a significant decrease in FWA before AF termination would have been demonstrated, especially in Lead V5. As discussed previously, this lead better displays LA electrical activity, and a decrease in FWA after ablation could be explained by the reduction of viable LA tissue.

The assessment of DF was carried out by means of atrial source separation, taking into account all ECG leads simultaneously, and it is unknown whether sequential single lead analyses using the Fourier Transformation method would have given comparable results.

Follow-up was performed in “real-life” settings by sequential 24-h Holter monitoring and symptoms assessment, but implantable loop recorders were not used.

Finally, LVA were defined by a bipolar voltage <0.25 mV, as suggested in a previous study [10], but no cardiac imaging—such as MRI—was performed for anatomical confirmation.

## 5. Conclusions

Our study suggests that FWA is not related to AF complexity but is mainly determined by the amount of viable atrial myocytes. Therefore, FWA should only be considered as a marker of atrial tissue pathology.

## Figures and Tables

**Table 1:** Clinical and procedural characteristics.

	*n* = 29
**Age (y)**	67.9 ± 9.2
**Female gender (*n*; %)**	8/29; 27.6%
**AF duration (months)**	21.7 ± 20.4
**Left atrial volume (mL)**	169.7 ± 37.1
**AF cycle length in LAA (ms)**	180.4 ± 24.3
**Number of LA dispersion-fragmentation zones per patient (*n*)**	5.5 ± 1.5
**Total LA surface of dispersion-fragmentation zones (cm^2^)**	21.3 ± 8.7
**Total LA surface of dispersion-fragmentation zones/Total LA surface (%)**	8.9 ± 3.6
**Right pulmonary vein ostium dispersion-fragmentation zone (*n*; %)**	26/29; 89.7%
**Left pulmonary vein ostium dispersion-fragmentation zone (*n*; %)**	26/29; 89.7%
**Anterior dispersion-fragmentation zone (*n*; %)**	21/29; 72.4%
**Left atrial appendage ostium dispersion-fragmentation zone (*n*; %)**	13/29; 44.8%
**Roof dispersion-fragmentation zone (*n*; %)**	19/29; 65.5%
**Septal dispersion-fragmentation zone (*n*; %)**	19/29; 65.5%
**Floor dispersion-fragmentation zone (*n*; %)**	6/29; 20.7%
**Lateral mitral isthmus dispersion-fragmentation zone (*n*; %)**	8/29; 27.6%
**Basal AF dominant frequency (Hz)**	5.31 ± 0.72
**Basal F-wave amplitude in lead I (µV)**	13.6 ± 5.7
**Basal F-wave amplitude in lead V1 (µV)**	47.4 ± 19.3
**Basal F-wave amplitude in lead V2 (µV)**	39.5 ± 13.0
**Basal F-wave amplitude in lead V5 (µV)**	23.9 ± 8.4
**AF termination during radiofrequency ablation procedure (*n*; %)**	19/29; 65.5%
**Follow-up duration (months)**	23.3 ± 9.8
**AF recurrence (*n*; %)**	2/29; 6.9%
**Recurrence of any atrial arrhythmia (*n*; %)**	13/29; 44.8%
**Follow-up duration to any atrial arrhythmia recurrence (months)**	4.0 [3.0–6.2]

## Data Availability

The datasets used and analyzed during the current study are available from the corresponding author on reasonable request.
